# Synergistic chemo-/photothermal therapy based on supercritical technology-assisted chitosan–indocyanine green/luteolin nanocomposites for wound healing

**DOI:** 10.1093/rb/rbac072

**Published:** 2022-09-26

**Authors:** Pei-Yao Xu, Ranjith Kumar Kankala, Yue-Wei Li, Shi-Bin Wang, Ai-Zheng Chen

**Affiliations:** Institute of Biomaterials and Tissue Engineering, Huaqiao University, Xiamen, Fujian 361021, PR China; Fujian Provincial Key Laboratory of Biochemical Technology (Huaqiao University), Xiamen, Fujian 361021, PR China; Institute of Biomaterials and Tissue Engineering, Huaqiao University, Xiamen, Fujian 361021, PR China; Fujian Provincial Key Laboratory of Biochemical Technology (Huaqiao University), Xiamen, Fujian 361021, PR China; Institute of Biomaterials and Tissue Engineering, Huaqiao University, Xiamen, Fujian 361021, PR China; Fujian Provincial Key Laboratory of Biochemical Technology (Huaqiao University), Xiamen, Fujian 361021, PR China; Institute of Biomaterials and Tissue Engineering, Huaqiao University, Xiamen, Fujian 361021, PR China; Fujian Provincial Key Laboratory of Biochemical Technology (Huaqiao University), Xiamen, Fujian 361021, PR China; Institute of Biomaterials and Tissue Engineering, Huaqiao University, Xiamen, Fujian 361021, PR China; Fujian Provincial Key Laboratory of Biochemical Technology (Huaqiao University), Xiamen, Fujian 361021, PR China

**Keywords:** photothermal therapy, supercritical carbon dioxide, antibacterial, antibiofilm, wound healing

## Abstract

Despite the success, it is highly challenging to battle against pathogenic biofilms-based chronic bacterial infections by conventional antibiotic therapy. Herein, we report a near-infrared (NIR)/acid-induced nanoplatform based on chitosan (CS)-coated indocyanine green (ICG, photosensitizer)/luteolin (LUT, a natural quorum sensing inhibitor) nanocomposites (ICG/LUT-CS) as antibacterial and antibiofilm agents for skin wound healing. Initially, the ICG/LUT nanoplatforms are prepared by the supercritical antisolvent technology and coated with the CS layer. The obtained ICG/LUT-CS with ultra-high encapsulation efficiency exhibited more favorable photothermal conversion effects and improved NIR laser/acid dual-induced drug release behavior than individual modalities, achieving exceptional bacteria-killing and biofilm elimination effects. Moreover, the ICG/LUT-CS realized the synergetic effects of chemotherapy and photothermal therapy outcomes for wound healing. Together, our findings provided an appealing strategy for the rapid preparation and future translational application of ICG/LUT-CS as an ideal agent for fighting against biofilm infections.

## Graphical Abstract

**Figure:**
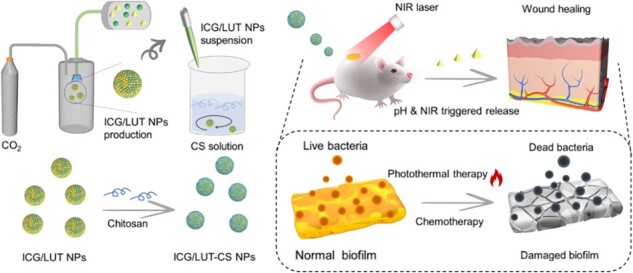

## Introduction

Pathogenic infections turned into chronic wounds have become critical public health issues associated with high morbidity and mortality [1]. Despite the success of antibiotics for antimicrobial therapy [2], the undeserved administration and misuse of antibiotics result in the emergence of drug-resistant bacteria and large-scale antibiotic pollution in the environment [3, 4]. More importantly, bacteria tend to be irreversibly adherent to the wound surface, promoting cell–cell communication pathways, producing extracellular polymeric substances and forming biofilms [5]. The emergence of biofilms substantially reduces the penetration of antibacterial agents and substantially immune response to bacteria, promoting drug tolerance and eventually leading to tissue damage [6, 7]. Therefore, it is urgent to develop an effective and safe strategy that can efficiently eliminate the threat of biofilms and remove all bacteria from the wound sites.

Recently, several advancements have evidenced novel antimicrobial and antibiofilm therapies, such as photothermal therapy (PTT) [8, 9], photodynamic therapy (PDT) [10] and chemodynamic therapy [11]. As one of the potent antibacterial and antibiofilm strategies photo-triggered thermal destruction offers enormous advantages, such as membrane disruption, permeability, protein/enzyme denaturation and inhibition of essential intracellular reactions, resulting in irreversible bacterial destruction [12, 13]. In addition, PTT exhibits significant benefits of minimal invasiveness, deep tissue penetration, imperceptible drug resistance and insignificant side effects [14, 15]. Despite the advantages and success, the high temperature (>70°C) for eradicating bacteria may sometimes cause severe damage to the adjacent healthy tissues, restraining the further application of PTT [16].

Several therapeutic approaches, such as traditional antibiotic treatment, PDT and chemodynamic therapy with efficient sterilization at a relatively lower temperature, have been integrated as an ideal strategy for achieving synergistic effects [17–20]. Previous reports indicated that combining PTT and small molecule drugs, including natural antibiotics and chemical synthetic antimicrobial agents, could enhance the penetration of agents into biofilms and inhibition of bacteria growth [21, 22]. The natural antimicrobial compounds in traditional herbal medicine have been considered the most promising therapeutic agents for synergistic bactericidal and antibiofilm effects [23]. Luteolin (LUT), a natural antibiotic, may induce lower drug resistance by destroying the cell membrane of bacteria, inhibiting nucleic acid synthesis, and interfering with protein expression, as well as energy metabolism [24]. Moreover, LUT acts as a quorum sensing inhibitor agent, which effectively inhibits the biofilm by attenuating the accumulation of relative signaling molecules, downregulating the gene expression and binding to the relative protein [25].

**Figure 1.**
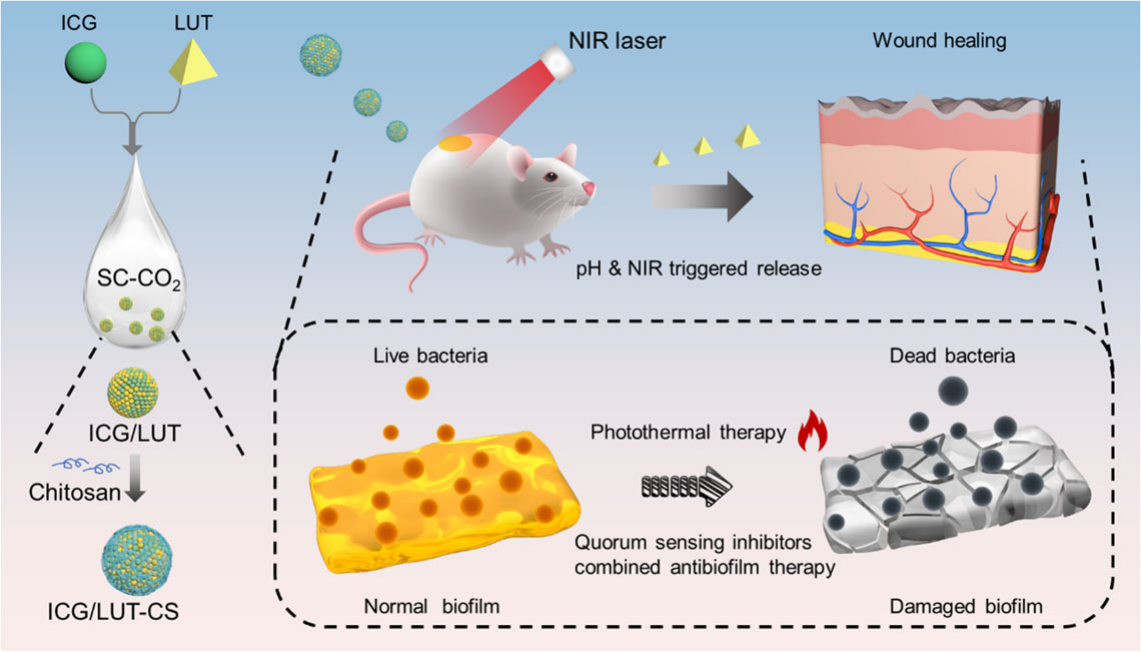
Schematic illustration of the development process of ICG/LUT-CS and synergistic PTT–chemotherapy for antibiofilm and antibacterial treatments.

Although the quorum sensing inhibitor agent has evolved as a promising weapon against infections, LUT suffers from inadequate bactericidal effect and poor solubility [26]. To address these limitations, nanoscale carriers were applied to improve their bioavailability toward achieving the bacteria-killing efficiency of LUT [27, 28]. Compared with free drugs, this nanoplatform could assist in biofilm eradication due to reducing drug absorption rate to extracellular polymeric substances, avoiding drug degradation in the biofilm and achieving sustained release of drug [29]. However, the low drug-loading capacity of those nanocarriers restricted their therapeutic efficacy. The controlled synthesis of nanoparticles with high loading content remained a challenge. Therefore, it is urgent to develop a multifunctional nanomedicine platform with high loading efficacy for synergistic antibacterial and antibiofilm therapy, as well as to reduce systemic side effects. The supercritical fluid (SCF) technology acts as a unique method for micro- and nano-size drug particle formation due to numerous advantages, generating carrier-free particles or drug-carrier composite particles for drug delivery in pharmaceutical applications [30–32]. Among various SCFs, supercritical carbon dioxide (SC-CO_2_) is the most frequently used solvent due to excellent characteristic features, including non-toxic, chemically inert, non-combustible, economical and recyclable [33, 34]. Indeed, SC-CO_2_ has been proven to be eco-friendly particle production technology due to moderate critical pressure and temperature, which would be suitable for industrialization [35, 36]. By considering these aspects, in this study, we demonstrate the fabrication of chitosan (CS)-modified indocyanine green (ICG) and LUT nanocomposites (ICG/LUT-CS) with promising traits *via* the supercritical antisolvent (SAS) approach and solution casting method. The innovation lies in the simple, fast and low-cost fabrication of nanocomposites toward addressing the challenges of synthesizing or modifying nanodrugs with high loading efficiency and integrating multiple functions in an “all-in-one” platform [37]. A mechanism based on photothermal-chemotherapy improved CS-based antibacterial activity to fight bacteria and biofilm under near-infrared (NIR) irradiation was proposed (Fig. 1). ICG/LUT-CS captured bacteria cells based on electrostatic interaction, which strengthened adhesion to the negatively charged surfaces of bacteria and enhanced the drug accumulation and penetration into biofilms. Under 808-nm laser excitation, ICG/LUT-CS performed excellent hyperthermia activity to damage cell integrity and reduced bacterial biofilms’ drug tolerance. Moreover, the photothermal effect significantly accelerated LUT release, damaging the intercellular protection system of bacteria and facilitating LUT delivery into biofilms. This synergistic PTT–chemotherapy eradication of bacterial and biofilm strategy displayed outstanding potential in reinforcing the therapeutic efficacy *in vivo* and *in vitro* and diminishing the side effects.

## Materials and methods

### Materials

ICG (purity of >95%), lysogeny broth (LB), and agar were obtained from Sangon Biotech. Co., Ltd. (Shanghai, China). LUT was purchased from Dalian Meilun Biotechnology Co. Ltd. (Dalian, China). CS (100–200 mPa.s, low Mol. Wt.), sodium chloride (NaCl) and ortho-nitrophenyl-beta-galactoside (ONPG) were purchased from Aladdin Co. Ltd. (Shanghai, China). Ethanol (EtOH), acetic acid, dichloromethane (DCM), dimethyl sulfoxide (DMSO) and thiazolyl blue tetrazolium bromide (MTT) were purchased from Sinopharm United Medical Device Co., Ltd (Beijing, China). CO_2_ was supplied by Xiamen Rihong Co., Ltd. (Xiamen, China). Mouse embryonic fibroblast (NIH/3T3) cells were obtained from the Type Culture Collection of the Chinese Academy of Sciences (Shanghai, China). A live/dead bacterial viability kit was purchased from Thermo Fisher Scientific (Waltham, USA). *Staphylococcus aureus* (ATCC 29213) was obtained from the China Center of Industrial Culture Collection (Beijing, China).

### Preparation of ICG/LUT-CS

Briefly, the SAS process was initially applied to prepare ICG/LUT, as reported previously [38]. ICG and LUT were dissolved in 14 ml of EtOH and added with 7 ml of DCM to obtain a homogeneous solution. To understand the influence of ICG and LUT ratio (2:1–1:2) in the SAS process, the nanonization of composites was performed under the same process conditions: Temperature = 40°C, pressure = 10 MPa, CO_2_ flow rate = 40 g/min and solvent flow rate = 1 ml/min. After attaining the stable predetermined conditions, the mixture solution was inducted into the SAS apparatus (Waters, Milford, USA) for particle formation. ICG/LUT constructs were dissolved in water and methanol solution, and the amount of ICG and LUT was analyzed by ultraviolet-visible-NIR (UV-vis-NIR) spectrophotometer at 780 and 361 nm and calculated according to the standard curve.

To immobilize CS molecules on the surface of ICG/LUT, 0.1 g of CS powder was dissolved in 100 ml of 2% acetic acid solution. The suspension of ICG/LUT (4 mg) in NaCl solution (1 M, 5 ml) was slowly added to the CS solution (5 ml) and stirred for 4 h. The ICG/LUT-CS were purified by centrifugation and washed twice with 1% acetic acid and thrice with dd-H_2_O. Afterward, ICG/LUT-CS constructs were immersed in the acetic acid solution for degrading the CS layer. In addition, water and methanol as solvents were used to extract the drug and measure the absorbance. The encapsulation efficiency (EE) of each drug was calculated as:

EE (%)=drug amount in nanoparticles/drug amount initially added×100.

### Characterizations

The surface morphologies of samples were measured by field emission-scanning electron microscopy at 5.0 kV (FE-SEM, SU5000, HITACHI, Tokyo, Japan). To further calculate the average size and particle size distribution, the diameters of randomly selected nanoparticles (*n* = 200) in SEM images were measured using the Nano Measurer (v1.2) software. The surface potential of samples was measured by a zeta potential analyzer (ZetaPALS, Malvern Panalytical Ltd., Malvern, England). UV-vis-NIR spectra of different samples were recorded on a spectrophotometer over a wavelength range of 300–1100 nm (Tu-1810, Beijing Purkinje General Instrument Co., Ltd., Beijing, China). Fourier transform infrared (FT-IR) spectra of samples were chronicled by attenuated total internal reflectance FT-IR (Nicolet iS50, Thermo Fisher Scientific, Waltham, USA) with a wave number range from 4000 to 400 cm^−1^. Powder X-ray diffractometer (PXRD) recordings were performed by Smar/SmartLa (Rigaku, Tokyo, Japan) to evaluate the crystalline change after SAS process.

### Photothermal effect measurement

To assess the photothermal effect of ICG/LUT-CS, 200 μl of dispersion in phosphate-buffered saline (PBS; 20, 40, 60 and 80 μg/ml) was irradiated by an NIR laser for 5 min. Meanwhile, the temperature increase behaviors of samples were recorded each 15 s interval by an NIR thermal imaging instrument (H16, Hikvision, Hangzhou, China).

### Drug release study

ICG/LUT-CS nanocomposites were initially dispersed in the PBS solution with different pH values (5.5 and 7.4). Furthermore, an NIR laser with 1.5 W/cm^2^ was irradiated. The mixture was kept be a shaker at around 150 rpm at 37°C for 48 h. One milliliter of supernatant was collected by centrifugation at the predetermined time and then supplied with 1 ml of the fresh medium. The concentrations of LUT were quantified at 351 nm against the corresponding standard curves of the drugs to determine the released amount of the drug.

### Antibacterial tests

*Staphylococcus aureus* strain, a typical bacteria, was used to investigate the synergistic PTT–chemotherapy antibacterial and antibiofilm activity. The bacteria were inoculated on LB and cultured in a 37°C shaker at 150 rpm for 12 h. Furthermore, part of the colony was suspended in the fresh broth and incubation for 8 h. To analyze the antibacterial efficacy of photoexcited ICG/LUT-CS, *S.aureus* was adjusted to 10^7^ CFU/ml with LB. Then, ICG/LUT and ICG/LUT-CS at the same concentration (ICG concentration = 5, 10, 20 and 40 μg/ml) were mixed with the bacterial solution. After irradiation for 5 min using an NIR light source with a power density of 1.5 W/cm^2^, the bacterial solution was incubated at 37°C for another 6 h. Subsequently, the treated bacterial solution was diluted by PBS, transferred and sprayed onto the LB nutrient agar plates. Visible colonies were then enumerated and photographed after incubation for 18 h at 37°C. The bacteria culture with LB treatment was used as a control. The colonies on the plate were counted using the Image J software.

### Biofilm inhibition assay

To investigate the biofilm inhibition activity of ICG/LUT-CS in comparison with ICG/LUT, the suspensions of nanoparticles were mixed with the bacterial suspension (10 μl, OD_600_ = 1) to obtain final concentrations of ICG (5, 10, 20 and 40 μg/ml) into a 96-well microtiter plate and irradiation for 5 min using NIR. The biofilm inhibition efficacy of different treatments was evaluated at the corresponding time intervals by the crystal violet (CV) method. In brief, the residual biofilm on the plate surface was obtained after washing twice with PBS, fixed with 95% EtOH, stained with 0.1% (w/v) CV solution for 10 min, washed and dried. The CV was dissolved using EtOH, and OD_550_ values were recorded with a microplate reader (Multiskan EX, Thermo Fisher Scientific, Waltham, USA).

### Antibiofilm eradication

To access the antibiofilm eradication activity, *S.aureus* (100 μl, 2 × 10^7^ CFU/ml) suspension was incubated for 48 h at 96-well plates. Then, the adherent bacterial biofilms grown on the surface were treated with different groups for 4 h. Afterward, the biofilms were irradiated by an NIR laser and then incubated at 37°C. The corresponding antibiofilm efficiencies of different groups and kinetic processes of antibiofilm were also evaluated by the CV staining method.

### Visualization of biofilm inhibition

*Staphylococcus aureus* biofilms were cultured on the cover glass, placed in a 24-well microtiter plate and treated with different groups, including bacteria treated with PBS as control. After treatment with NIR laser, residual biofilms were washed with PBS twice and stained with a live/dead bacterial viability kit for 20 min. Furthermore, the fluorescent images of bacteria in biofilms were captured using fluorescence microscopy (Axio Observer 3, Zeiss, Oberkochen, Germany), and ablation effects were also observed using FE-SEM. In brief, the residual biofilms were treated as described above, washed with PBS, fixed with 2.5% glutaraldehyde at 4°C overnight, then dried with different ethanol grades (20, 40, 60, 80 and 100%), respectively. Finally, the morphological transformation of *S.aureus* in biofilms was characterized according to the standard specification.

### Bacteria membrane permeability measurement

The ONPG hydrolysis assay was performed to characterize the leakage of the bacterial membrane. After being irradiated by NIR, *S.aureus* strain in the biofilms was mixed with ONPG solution and incubated for 24 h. After treatment, OD_420_ values were recorded with a microplate reader.

### *In vivo* antibacterial and wound-healing analysis

For *in vivo* antibacterial assays, male Balb/c mice (8–10 weeks) with an average weight of 25 g were used to establish the wound infection model. All animal studies were performed under the guideline approved by the Institutional Animal Care and Use Committee of Huaqiao University and following the Administration of Affairs Concerning Experimental Animals of China. Mice were randomly divided into five groups: (i) PBS, (ii) ICG/LUT, (iii) ICG/LUT+NIR, (iv) ICG/LUT-CS and (v) ICG/LUT-CS+NIR for the wound-healing experiments. Full-thickness wounds with 0.5 cm diameter were grafted on the back of the mice, and *S.aureus* suspension (10^8^ CFU/ml, 50 μl) was inoculated on the wound area for 24 h.

The dispersions of different samples (ICG concentration = 40 μg/ml, LUT concentration = 20 μg/ml, 50 μl) were sprayed carefully on the infected wounds and irradiated for 5 min with an NIR laser (1.5 W/cm^2^). The real-time temperature of the infection wound was captured by an NIR thermal imaging camera. In addition, the effect of treatment was evaluated every 2 days by recording the wound area using a digital camera. The wound area was measured by tracing around the margin *via* Image J software. The percentage of wound closure was calculated as:

$$\text{Wound area}\;\left(\%\right)=S_{\text{experiment}}/S_{\text{day}\;0}\times 100.$$

For analyzing *in vivo* antibacterial activity, infected wound exudate was taken every 2 days and diluted with PBS. Furthermore, the *S.aureus* suspension was incubated and cultured in a 37°C shaker at 150 rpm for 4 h, and the OD of the medium at 600 nm was measured by a UV-vis-NIR spectrophotometer. In addition, 100 μl of diluted *S.aureus* suspension was spread on the LB agar plate for 24 h, and digital photographs of the colonies were taken.

After 8 days of treatment, the wounded tissues from euthanized mice were harvested, fixed with 4% paraformaldehyde and stained with hematoxylin and eosin (H&E) embedded in paraffin. The wound tissues were also stained with Masson’s trichrome for wound-healing evaluation. All histological images were captured using an optical microscope.

### Biocompatibility

The NIH/3T3 cells were used to evaluate the biocompatibility of materials *in vitro*. 1 × 10^4^ cells were seeded in the 96-well plates and then cultured at 37°C for 24 h in dulbecco’s modified eagle medium containing 10% fetal bovine serum. Then, after incubation with different samples (same concentration gradient) for 30 min, an NIR laser at an intensity of 1.5 W/cm^2^ was irradiated for 5 min and incubated for 24 h. After that, cells were washed with PBS and treated with a medium containing MTT (10%, 200 μl) for 4 h. Then, the working solution was removed and added with DMSO to dissolve the formazan crystals. Finally, OD_570_ values were determined by a microplate reader. NIH/3T3 cells treated with a culture medium were used as control. The cell survival was calculated as:

$$\text{Cell survival}\;\left(\%\right)={\;\text{mean OD}}_{\text{sample}}\;/\;{\text{mean OD}}_{\text{control}}\times 100.$$

For testing the hemolysis rate *in vitro*, the ICG/LUT-CS was mixed with 4% (v/v) human erythrocyte suspension to final concentrations at 30, 60, 120 and 240 μg/ml and incubated at 37°C for 30 min. Then, the mixture was centrifuged, and the absorbance of supernatants at 570 nm was determined using a UV-Vis spectrophotometer. The hemolysis percentage of ICG/LUT-CS *in vitro* was calculated as (PBS and distilled H_2_O treatment as the negative control and positive control groups, respectively):

$$\text{Hemolysis}\;\left(\%\right)=({\text{OD}}_{\text{sample}}–{\text{OD}}_{\text{negative control}})/({\text{OD}}_{\text{positive control}}–{\text{OD}}_{\text{negative control}}).$$

To test biocompatibility *in vivo*, the body weights were measured every 2 days. The blood samples from the sacrificed mice were subjected to analyze liver and kidney function tests. In addition, the major organs were dissected, fixed, embedded and stained with H&E.

## Results and discussion

### Optimization of SAS parameters

To further demonstrate the successful preparation of the ICG/LUT, the morphologies of particles were characterized by FE-SEM. Accordingly, the drug ratio significantly influenced the SAS-based particle formation process [39]. The SEM images of raw LUT and ICG/LUT showed that the unprocessed LUT showed irregular needle-shaped crystals, which could be due to insoluble phenolic components with different ratios (see Supplementary Fig. S1). To this end, the ICG/LUT composites were precipitated in the forms of nanoparticles and nanowires at the predetermined ratio of 1:2, indicating the discrepancy between nucleation and growth rates in ICG and LUT [40]. With the increasing ratio of ICG and LUT to 1:1, the resultant particles tended to be aggregated. Interestingly, in the case of an increase in ICG amount to the ratio ICG/LUT of 2:1, the resultant particles produced by SAS showed nano-sized spherical structures. The drastic changes in the morphology of ICG/LUT particles could be based on the droplet formation rate that would outstrip the nucleation rate with a decrease in the concentration of LUT [41].

**Figure 2.**
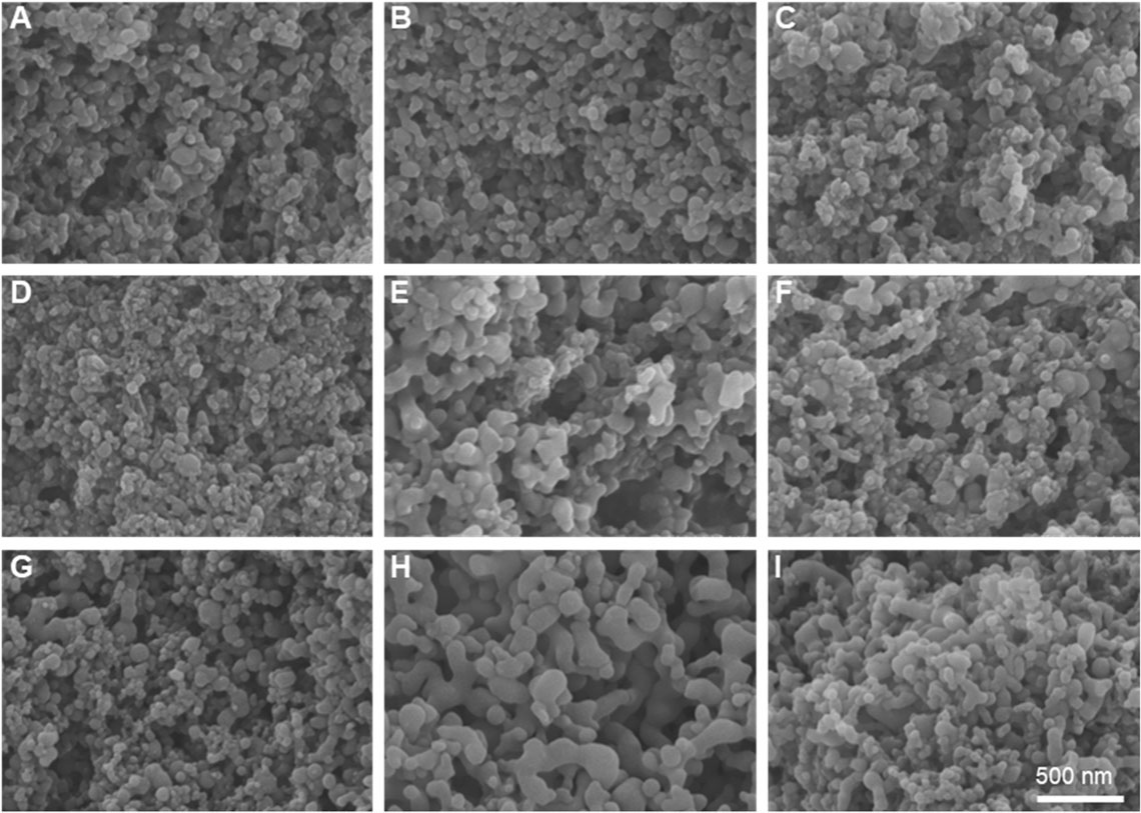
SEM images of ICG/LUT were obtained by the SAS process in different run orders, as shown in Supplementary Table S1. (**A**) 40 g/min—12 MPa—1 ml/min, (**B**) 30 g/min—10 MPa—1 ml/min, (**C**) 30 g/min—12 MPa—0.5 ml/min, (**D**) 40 g/min—12 MPa—0.5 ml/min, (**E**) 40 g/min—10 MPa—0.5 ml/min, (**F**) 30 g/min—12 MPa—1 ml/min, (**G**) 40 g/min—10 MPa—1 ml/min, (**H**) 30 g/min—10 MPa—0.5 ml/min and (**I**) 35 g/min—11 MPa—0.75 ml/min, scale bar = 500 nm.

To further understand the relationship between operating conditions with ICG/LUT, a 2^3^ Minitab-based factorial method was applied as a suitable tool for calculating and producing ICG/LUT. The surface morphology of ICG/LUT under different operation conditions was obtained by SEM (Fig. 2). The quantitative analysis of the average size and span of ICG/LUT produced at the operating parameters for experimental results were presented (see Supplementary Table S1).

To compare the impact of the operating factors and their interactions on the particle size and span, a Pareto chart, normal plot, main effect plot and interaction plot were presented (see Supplementary Fig. S2) to explore the effects of significant variables in the SAS process. Except for the quadratic effect of the flow rate of CO_2_ and pressure, all factors with a positive impact on particle size and the interaction between each other were independent. Among these, an increase in pressure, as well as flow rates of CO_2_ and solution, showed significant effects on the eventual particle size, emphasizing that the supersaturation and mass transfer significantly affected the SAS-assisted particle formation [42]. In terms of span, the combined effect of pressure and solution flow rate is statistically significant, indicating the difficulty in controlling the span due to the complex particle formation mechanism in the SAS process (see Supplementary Fig. S3).

Moreover, the ICG/LUT samples showed monodisperses spherical structure (see Supplementary Fig. S4) after ultrasonication, confirming the successful production of nanoparticles with good dispersion by the SAS process. Furthermore, the PXRD patterns of samples were recorded to present the crystalline features of the designed samples (see Supplementary Fig. S5). The physical mixture of LUT and ICG showed the multiple typical peaks of LUT at 14.3°, 24.5°, 26.3°, 27.1° and 28° [43], which were lacking in the ICG/LUT. The absence of such crystalline peaks generally indicates the amorphous nature, which is suitable for improving the solubility of the drug compared to its raw form of LUT. The principal reason could be decreased time for nuclear growth due to rapid mass transfer during the SAS process, inducing the formation of ICG and LUT with an amorphous structure [44].

### Characterizations of ICG/LUT-CS

**Figure 3.**
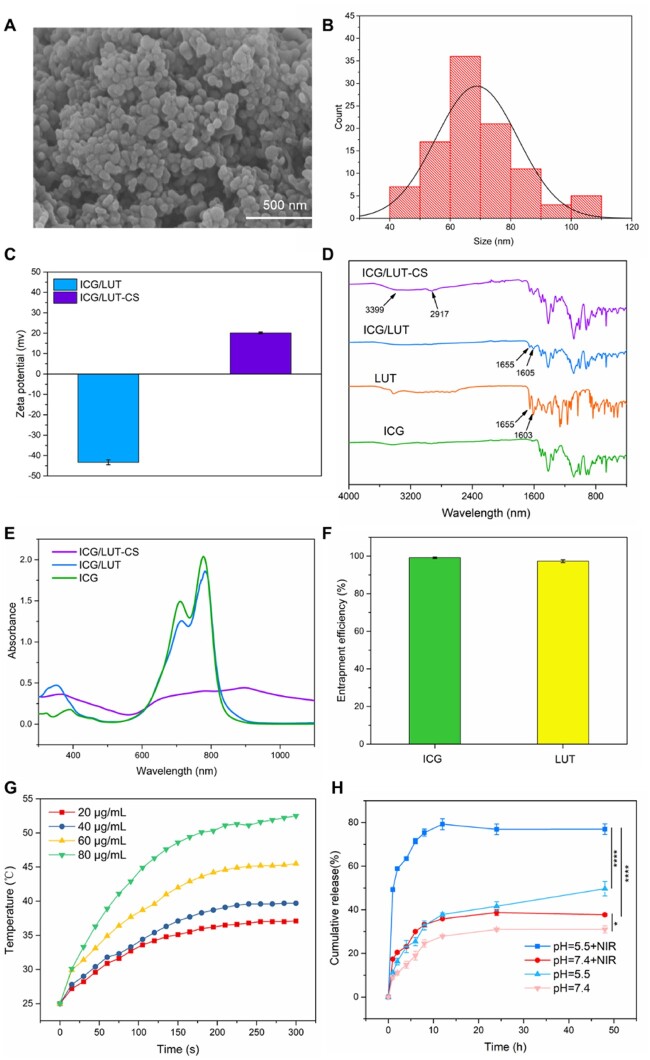
Physicochemical properties of ICG/LUT-CS. (**A**) SEM photograph of ICG/LUT-CS, scale bar = 500 nm, (**B**) the size distribution of ICG/LUT-CS, (**C**) zeta potential, (**D**) FT-IR spectra, (**E**) UV-vis-NIR spectra, (**F**) EE of ICG/LUT-CS, (**G**) photothermal heating curves of ICG/LUT-CS in different concentrations and (**H**) *in vitro* cumulative release profiles of LUT in different conditions.

Due to intrinsic bacteria-killing ability, CS-based nanomaterials driven by electrostatic interactions or hydrogen bond linkages have been used extensively in wound dressings and antibacterial applications [45]. In this framework, CS was chosen as a capping reagent for improving bacteria-killing ability and preventing drug leakage. As shown in Fig. 3A and B, the coating of CS over the ICG/LUT still maintained the spherical and uniform structure of the latter components with an average particle size of 64.4 nm and exhibited a narrow size distribution. Subsequently, the zeta potential of the particle shifted to the opposite charge, confirming that CS was successfully coated on the surface of the ICG/LUT (Fig. 3C). It was observed from the FT-IR spectrum of ICG/LUT coprecipitates that most of the chemical groups replicated with the ICG spectrum, indicating no significant influence of the SAS process on the chemical functionalities of the drugs (Fig. 3D). Compared with ICG molecules, the characteristic of carbonyl stretching (1615 and 1655  cm^−1^) from the central heterocyclic ring signals could be observed in ICG/LUT, verifying the existence of ICG and LUT in the coprecipitates [27]. After coating with CS, the broadband at 3399 and 2917 cm^−1^ could be observed, attributing to the O−H stretching vibration and the C−H stretching of CS, respectively [46, 47].

Moreover, the UV-vis-NIR spectra (Fig. 3E) revealed that there were no significant differences in the strong absorption peaks with the pure drug molecules in terms of the intensity of around 780 nm between ICG/LUT and ICG, a broad adsorption peak ranging 350 nm in ICG/LUT, indicating the existence of LUT and ICG [48]. After being coated with CS, a broader absorption band displayed in the range of 600–1000 nm, indicating the electrostatic interactions between ICG and CS [49]. More importantly, the EE of ICG and LUT was as high as ∼98.4% and ∼97.7%, respectively (Fig. 3F), indicating that the layer-by-layer process could substantially minimize the drug loss compared to other loading procedures. The photothermal conversion curve demonstrated that the temperature with the total concentration at 80 μg/ml sharply increased and reached 55°C after irradiation for 5 min (Fig. 3G). Notably, the temperature of ICG/LUT-CS suspension increased with irradiation time and ICG concentration, demonstrating the successful preservation of the NIR-induced photothermal properties of ICG after the SAS process and coated with CS [50].

Furthermore, the LUT release from ICG/LUT-CS was explored *in vitro* under different conditions (Fig. 3H). It was observed that LUT molecules were released significantly higher at pH 5.5 compared to pH 7.4. The improved drug release behavior happened to be favorable due to the acid-responsive behavior of CS, facilitating LUT release [51]. Moreover, the thermal-response property of ICG was also harnessed to endow the nanocomposites with NIR-induced drug release properties. These pH/NIR-responsive intelligent nanocomposites presented a more rapid release of LUT with around 80% release after 24 h in the acidic microenvironment and NIR stimulation. These pH- and light-responsive release findings indicated that the designed composites offer the potential for further antibacterial and antibiofilm activities based on PTT treatment.

### *In vitro* antibacterial study

**Figure 4.**
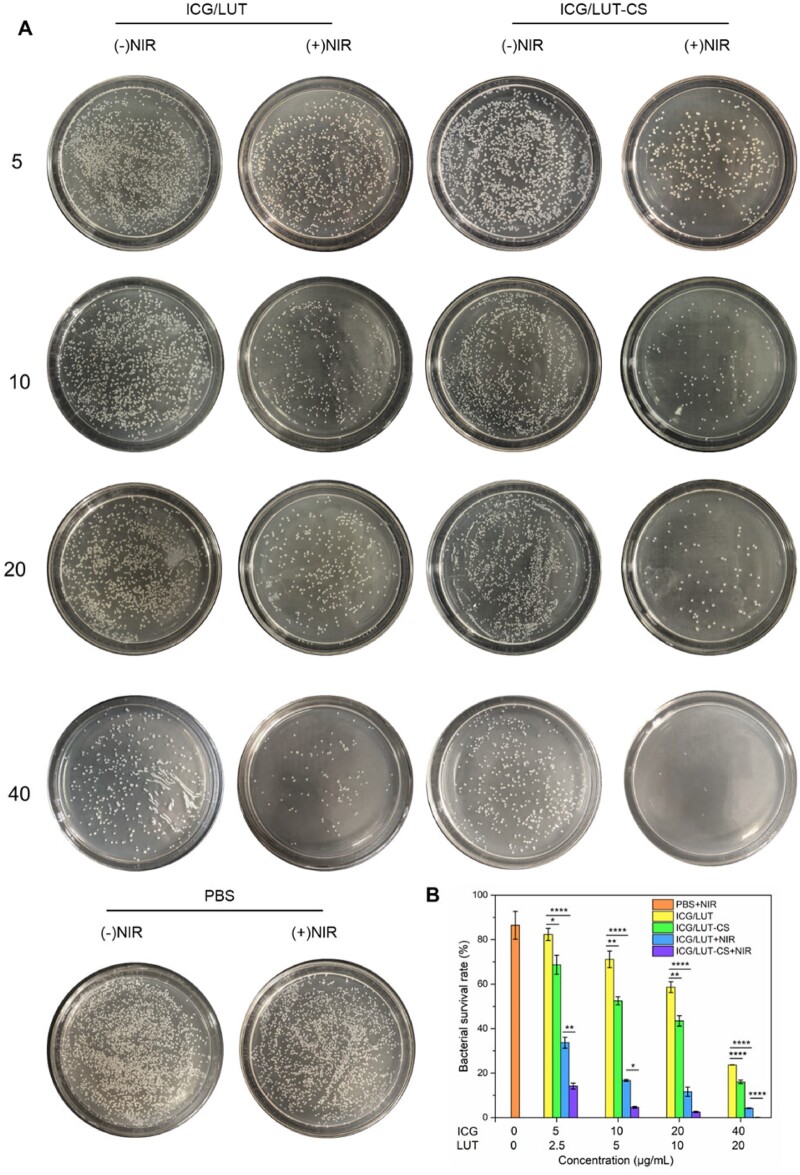
*In vitro* antibacterial performance of ICG/LUT-CS, (**A**) images of *S.aureus* bacterial colonies and (**B**) the corresponding bacterial survival rates grown on broth agar plates after different treatments.

Initially, we evaluated the antibacterial efficacy of different samples against typical, highly infectious gram-negative bacteria (*S.aureus*) *in vitro*. As shown in Fig. 4, NIR application alone displayed no significant effect on *S.aureus* growth on plates compared to the control treatment group (media alone). Different drugs showed concentration-dependent bacteria-killing efficiency. The SAS-assisted ICG/LUT composite displayed limited bactericidal outcome in the absence of NIR, which might be due to the partly bacteriostatic activity of LUT at the therapeutic range. Interestingly, the subsequent ICG/LUT-CS composites treatment group showed 50% bacteria reduction at a concentration of 10 µg/ml. Remarkably, in the presence of the NIR treatment, the bactericidal effects of ICG/LUT and ICG/LUT-CS against *S.aureus* were significantly increased. Notably, the antibacterial rate of ICG/LUT-CS could reach almost 100% at a concentration of 40 µg/ml, indicating the strengthened PTT-based bactericidal effect.

### *In vitro* antibiofilm study

**Figure 5.**
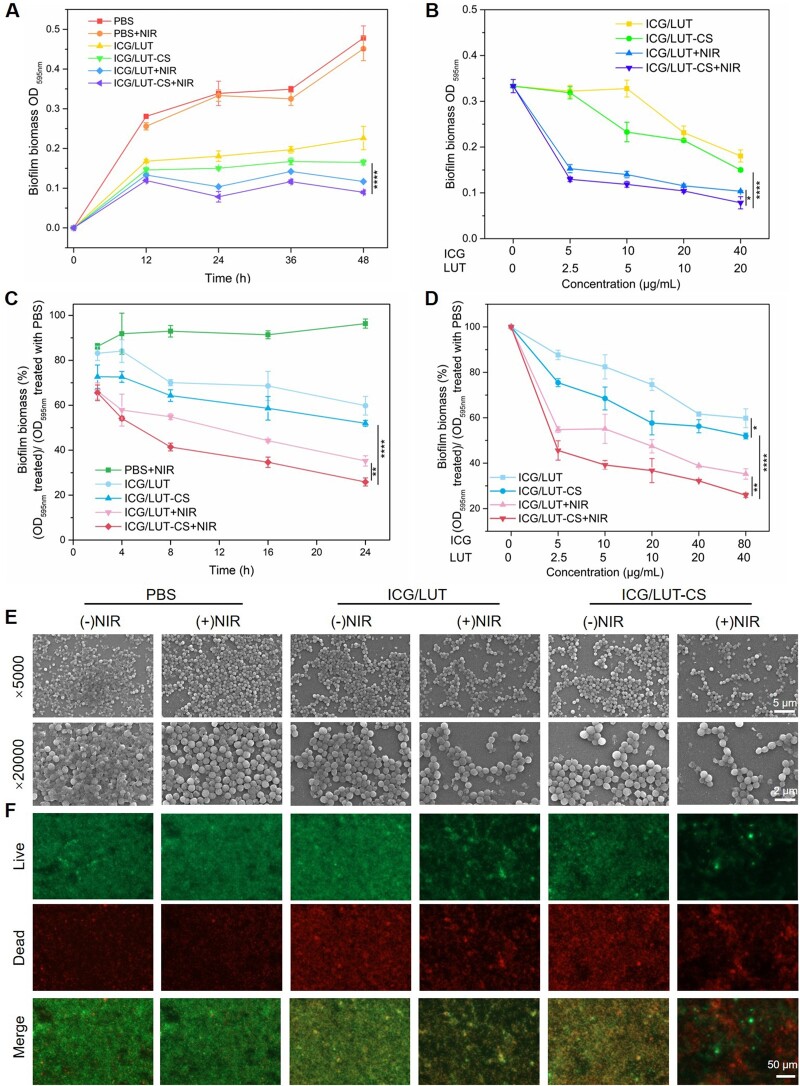
*In vitro* inhibition effect of *S.aureus* biofilm formation and *S.aureus* biofilm ablation performance of ICG/LUT-CS, (**A**) quantitative analysis of the CV-stained biofilms with different pretreatments for different times, (**B**) pretreatments with different concentrations of nanoparticles for 24 h, (**C**) relative *S.aureus* biofilm biomass after pretreatments with different concentrations of nanoparticles for 24 h, (**D**) relative the remaining *S.aureus* biofilm biomass with different incubation time, (**E**) relative the remaining *S.aureus* biofilm biomass with different concentrations of nanoparticles for 24 h, (**F**) membrane permeability of remaining *S.aureus* biofilms cells, (**G**) SEM images (scale bar = 5 and 2 μm) and (**H**) live/dead staining images of remaining *S.aureus* biofilms with different treatments (scale bar = 50 μm).

Biofilms are referred to as extracellular polymeric structures related to human infectious diseases to improve antibiotic tolerance and resistance to microorganisms. Owing to their highly protective nature, it is vital to develop novel antibacterial strategies that inhibit biofilm formation and eradicate formed biofilms. In this study, the inhibition effect of the nanoplatform on *S.aureus* biofilm formation was examined and quantified by the CV staining assays. As depicted in Fig. 5A, the biofilm growth was time-dependent, and only the NIR treatment group displayed the same growth tendency compared with the PBS group, indicating that NIR showed no effect on biofilm formation. In the absence of NIR laser, ICG/LUT and ICG/LUT-CS treatment groups showed a particular antibiofilm effect, demonstrating the responsive release and antibiofilm effect of LUT. Notably, the inhibition of biofilm growth was also concentration-dependent (Fig. 5B). More importantly, ICG/LUT-CS group with NIR laser irradiation (ICG concentration = 40 µg/ml) showed augmented inhibitory ability on biofilms than ICG/LUT-CS treatment group.

In addition, the ability to eradicate established biofilms is a valuable index to examine the efficiency of antibacterial therapeutics. After quantitative analysis of the remaining biomass in 24 h (Fig. 5C), the therapeutic effect of nanoplatform against *S.aureus* biofilm increased gradually with concentration. In contrast, the antibiofilm effect of ICG/LUT-CS in the presence of NIR irradiation was substantially higher at all tested concentrations. For instance, at an ICG concentration of 80 µg/ml, ICG/LUT-CS+NIR exhibited more antibiofilm activity against *S.aureus* (74.1%) compared with the PBS+NIR group (3.7%), ICG/LUT (40.2%), ICG/LUT-CS (48.0%) and ICG/LUT+NIR (64.78%). These experimental results showed synergistic antibiofilm effects in hyperthermia, nanoparticle–biofilms interaction and responsive release of LUT. Furthermore, the biofilm inhibiting effects with various treatments for different incubation times were also quantitatively measured by CV staining assay (Fig. 5D). Although they induced time-dependent biofilm inhibition, the designed nanoplatforms resulted in a poor antibiofilm effect, indicating the limitation of chemotherapy. However, it was worth noting that the NIR-induced antibiofilm effects increased sharply in 8 h. Then, the trend became smooth, which could be attributed to NIR-induced high temperature and LUT release.

To further qualitatively evaluate the biofilm elimination effect, SEM images of the treated biofilms were captured. As illustrated in Fig. 5E, the spherical shape *S.aureus* cells were clumped together and resulted in biofilm with dense reticular structure. Upon ICG/LUT and ICG/LUT-CS treatment, the adhesive bacteria of biofilm were reduced. However, *S.aureus* showed negligible structural changes, suggesting the limited chemotherapy effect on *S.aureus* biofilm. Nevertheless, ICG/LUT-CS+NIR treatment group showed apparent elimination of *S.aureus* biofilms, and cells were morphologically deformed, indicating that PTT treatment and chemotherapy exhibited synergistic antibiofilm activity and improved the bacteria-killing efficiency. Furthermore, the microscopic fluorescence observations were presented to visualize the chemotherapy–photothermal biofilm eradication effect. As shown in Fig. 5F, the dense stacking biofilm structure with live *S.aureus* cells remained in a glass dish with PBS+NIR treatment, specifying the invalidity of only treatment with NIR. In addition, the nanoparticles without NIR treatment only led to a slight effect on *S.aureus* cell-killing efficiency based on the antibiofilm and bacteria-killing property of LUT, further confirming that the adhesive biofilm significantly decreased the antibiotic sensitivity of these bacterial cells. The ICG/LUT with NIR treatment damaged the dense stacking structure of biofilm and presented a large amount of red fluorescence, indicating the effectiveness of the hyperthermia effect for heightening the bactericidal action based on LUT alone. Strikingly, the *S.aureus* biofilm was eliminated, and the green fluorescence was decreased upon ICG/LUT-CS with NIR treatment, revealing that CS modification on nanoparticles improved the chemotherapy–photothermal synergistic effect for complete elimination of bacterial biofilm.

Then, the ONPG hydrolysis assay was used to analyze the degree of cell membrane damage of *S.aureus* (see Supplementary Fig. S6) [52]. Under chemotherapy–photothermal synergistic treatment, the content of intracellular β-d-galactosidase in bacteria significantly increased compared to that in the chemotherapy alone and control group, suggesting the PTT treatment efficacy in damaging the *S.aureus* membrane. Compared to ICG/LUT+NIR treatment, the membrane permeability of *S.aureus* was remarkably increased in ICG/LUT-CS+NIR, implying that membrane destruction was significantly aggravated by CS-modified nanoparticles due to their improved penetration into *S.aureus* biofilm easily and deliver ICG and LUT [53].

### *In vivo* assessment of wound healing

**Figure 6.**
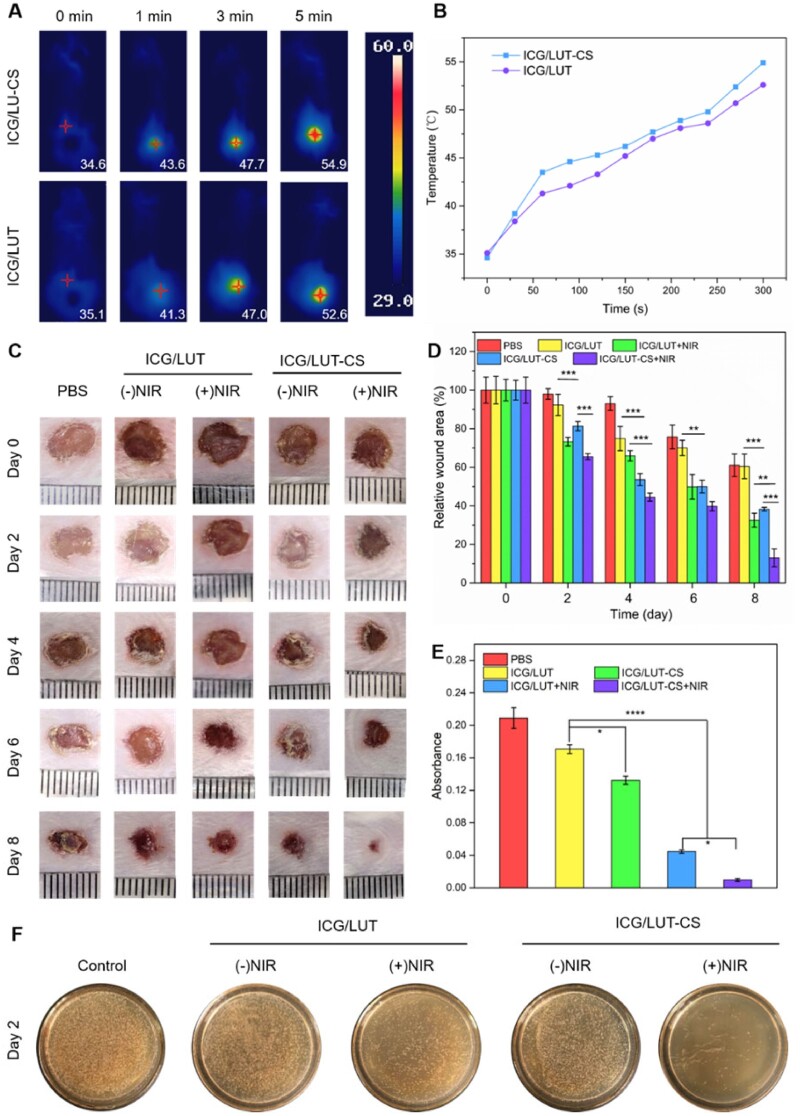
Antibacterial and wound-healing performance of ICG/LUT-CS *in vivo*. (**A**) *In vivo* thermal imaging of *S.aureus*-infected skin wound treated at different time intervals, and (**B**) the corresponding temperature variation of wound site after treated with ICG/LUT and ICG/LUT-CS upon 808-nm laser irradiation, (**C**) representative images of wound, (**D**) quantitative data of wound area, (**E**) OD of the different groups after collection form wound site and 4 h incubation on Day 2, (**F**) photographs of *S.aureus* colonies isolated from wound site grown on broth agar plates after receiving various treatments on Day 2.

Encouraged by the attractive antibacterial and antibiofilm properties of ICG/LUT-CS *in vitro*, the synergistic effect of this nanoplatform was further explored *in vivo* by *S.aureus*-infected skin wound model. As shown in Fig. 6A, the temperature of *S.aureus*-infected skin wound increased fast within 300 s under NIR irradiation. The wound region temperature of the mouse in the ICG/LUT and ICG/LUT rapidly increased from 35°C to 52.6°C and 54.9°C, respectively (Fig. 6B). The phenomenon provided an intuitive proof of the PTT effect of ICG/LUT and ICG/LUT-CS *in vivo*, proving that this constructed nanoplatform acted as an attractive therapeutic agent based on the PTT effect for bacteria-killing and promoting wound-healing effects.

Based on the superior antibacterial and antibiofilm activity, we attempted to investigate the antibacterial efficacy and wound healing *in vivo* by creating *S.aureus*-infected full-thickness excisional wounds. The macroscopic investigations revealed that the fresh granulation tissue regenerated around the wound area with combination treatment and showed no signs of ulceration and suppuration. In addition, ICG/LUT-CS with the NIR group exhibited a superior wound-healing rate and a more remarkable effect on wound area reduction (Fig. 6C). According to the quantitative analysis of wound areas (Fig. 6D), the wound-healing ability of combinational chemo-photothermal treatment was tremendously improved compared to the single-therapy groups. Impressively, the remaining wound area percentage of ICG/LUT-CS+NIR treatment after 8 days was 13.1 ± 4.7%, significantly lower related to ICG/LUT+NIR treatment (38.3 ± 1.0%), indicating that CS modification greatly accelerates the wound-healing effect. The growth of bacteria in the wound areas could be estimated clearly *via* culturing (Fig. 6E and F) and counting the recultivated bacterial colonies. On Day 2, compared with the control group, the OD_600_ values of ICG/LUT and ICG/LUT-CS treatment groups were decreased to 0.170 ± 0.005 and 0.132 ± 0.005, respectively. Moreover, almost all residual live bacteria in wounds were cleaned during ICG/LUT-CS+NIR treatment (OD_600_ = 0.010 ± 0.002), and the unsurpassed antibacterial activity may benefit from the enhanced chemotherapy/PTT (see Supplementary Fig. S7).

**Figure 7.**
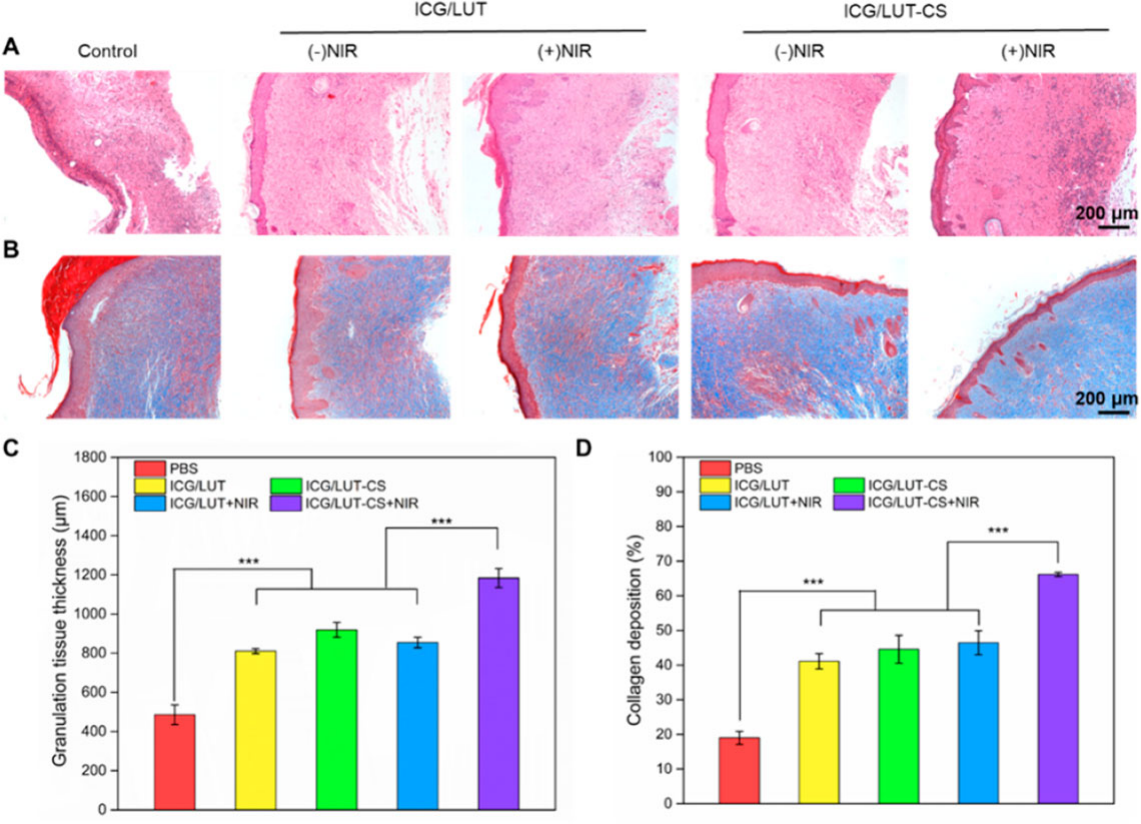
Histological evaluation of wounds in different groups. (**A**) H&E staining of infected skin wound tissues, (**B**) Masson staining of infected skin wound tissue (scale bar = 200 μm), (**C**) the corresponding quantitative data of granulation tissue thickness and (**D**) collagen deposition.

In addition, the regenerated skin tissues around the infected wound area were determined by H&E staining and Masson staining. On Day 8, epithelial layers were covered on the wound tissue in all the treatment groups. However, the apparent infiltration of inflammatory cells was observed in the control group, indicating a severe bacterial infection. In contrast, the ICG/LUT-CS+NIR group contained fewer inflammatory cells, new hair follicles and blood vessels formed (Fig. 7A). Remarkably, the wounds treated with ICG/LUT-CS+NIR group showed the thickest and highest regularity granulation tissue (*P* < 0.001) compared with the wounds treated with other groups (Fig. 7C). To this end, the Masson staining demonstrated that ICG/LUT-CS treated wounds displayed more collagen deposition and fiber alignment than healthy skin (Fig. 7B and D). In short, the ICG/LUT-CS-based synergistic bacterial killing effect could be promising for promoting epidermal remodeling and speeding up the wound-healing repair process.

### Biocompatibility

**Figure 8.**
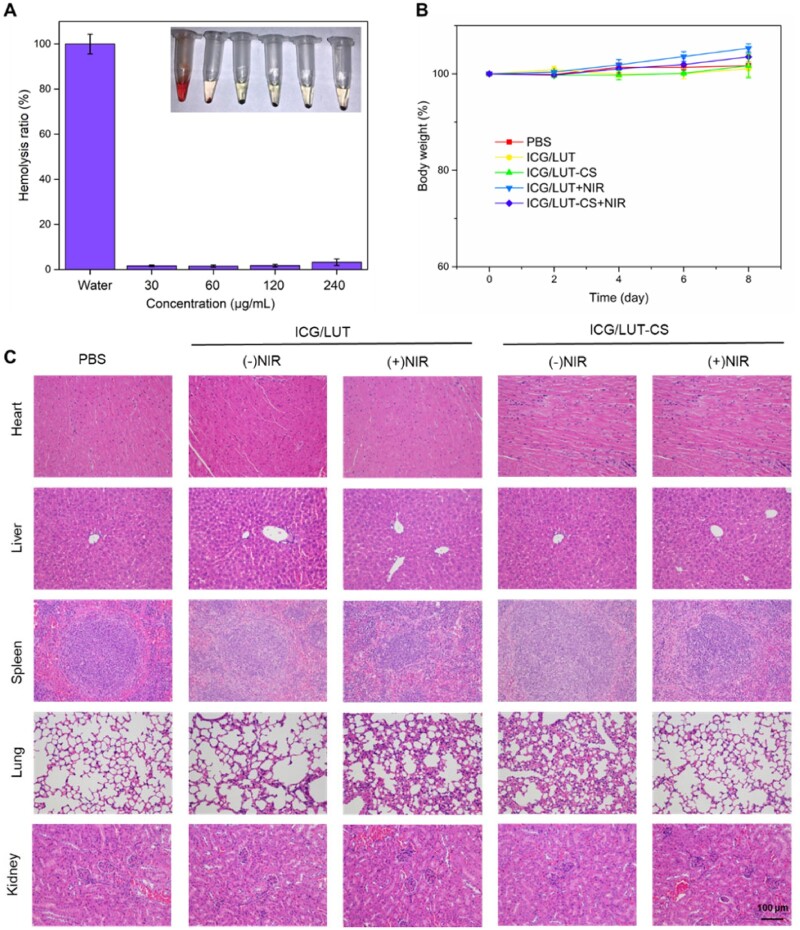
Biosafety assessment of ICG/LUT-CS, (**A**) hemolysis ratio and corresponding photographs of whole blood after treatment with different concentrations of ICG/LUT-CS, (**B**) weight change curves in *S.aureus*-infected mice after different treatments from Day 0 to 8 and (**C**) H&E staining of major organs after 8 days of treatment (scale bar = 100 μm).

The cytotoxicity of ICG/LUT and ICG/LUT-CS under NIR treatment *in vitro* was further evaluated using NIH/3T3 cells (see Supplementary Fig. S8). It was found that there was no apparent cytotoxicity on these cells treated with the designed nanoplatforms *in vitro*. The cell survival rate of NIH/3T3 cells remained over 80% under treatment dose, indicating the obtained nanoplatforms with good biocompatibility for treating bacterial infections. The hemolysis rate of ICG/LUT-CS at different concentrations was shown in Fig. 8A. The ICG/LUT-CS-treated group showed no significant hemocyte breakage when ICG/LUT-CS concentration was up to 240 μg/ml, suggesting excellent hemocompatibility of ICG/LUT-CS. The changes in the body weight during the treatment period, the morphology of major organs, and blood biochemical after different treatments were evaluated to assess the biocompatibility of ICG/LUT-CS. The average body weight of the mice with different treatments showed no apparent changes (Fig. 8B), indicating that ICG/LUT-CS possessed negligible adverse effects during the antibacterial therapy process. Furthermore, the H&E staining of all tissues showed typical morphological characteristics, reflecting that there was no damage to the major organs of the mice (Fig. 8C). The blood routine examination and biochemical parameters results showed no significant difference in the levels of these treatment groups compared to the control group, indicating an excellent hepatic and liver safety of these nanoplatforms (see Supplementary Fig. S9). Together, these findings clearly illustrated that ICG/LUT-CS possessed excellent biocompatibility, which could serve as an active antibacterial nanomaterial for synergistic anti-infective therapy.

## Conclusion

In summary, a versatile chemotherapy–photothermal antibacterial nanoplatform (ICG/LUT-CS) was developed for the synergistic treatment of *S.aureus*-infected skin wounds. ICG/LUT-CS was fabricated using the combination of SAS technology and solution casting method, which displayed a spherical and uniform structure with monodisperse and superior NIR-driven temperature rising performance. Compared to the chemotherapy process or PTT treatment alone, the combination of NIR-induced hyperthermia with the release of LUT for chemotherapy and CS-induced antibacterial activities could significantly eradicate bacteria and inhibit biofilm *in vitro*. Notably, *in vivo* wound-healing experiments demonstrated that NIR-driven ICG/LUT-CS nanoplatform could synergistically eliminate the *S.aureus* infection and accelerate the wound-healing process. In conclusion, this newly developed antibacterial nanoplatform with excellent biocompatibility provided an effective and promising way of alleviating bacterial infections.

## Supplementary data

Supplementary data are available at *REGBIO* online.

## Funding

This work was supported by the National Key Research & Development Program of China (2019YFE0113600); the National Natural Science Foundation of China (NSFC 81971734, 32071323); and Program for Innovative Research Team in Science, Scientific Research Funds of Huaqiao University (21BS113).

*Conflicts of interest statement*. None declared.

## Supplementary Material

rbac072_Supplementary_Data
