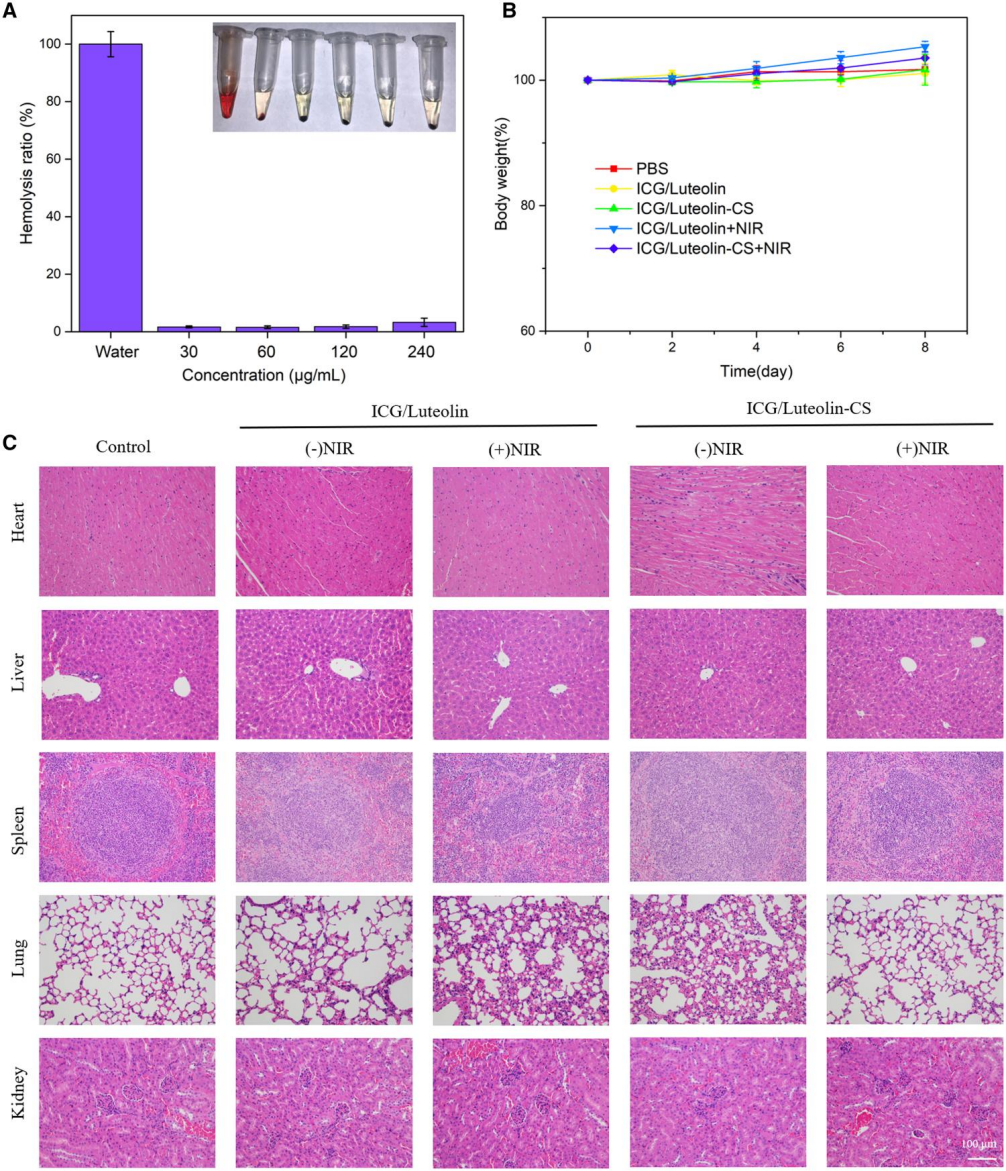# Correction to: Synergistic chemo-/photothermal therapy based on supercritical technology-assisted chitosan–indocyanine green/luteolin nanocomposites for wound healing

**DOI:** 10.1093/rb/rbae037

**Published:** 2024-04-09

This is a correction to: Pei-Yao Xu, Ranjith Kumar Kankala, Yue-Wei Li, Shi-Bin Wang, Ai-Zheng Chen, Synergistic chemo-/photothermal therapy based on supercritical technology-assisted chitosan–indocyanine green/luteolin nanocomposites for wound healing, *Regenerative Biomaterials*, Volume 9, 2022, rbac072, https://doi.org/10.1093/rb/rbac072

Figure 8 of this article contained an accidental duplication in the HE images of heart (ICG/Luteolin-CS (-NIR) and ICG/Luteolin-C (+NIR)) and the liver (PBS and ICG/Luteolin-CS (-NIR)). A corrected version of the figure appears below.

These details have been corrected only in this correction notice to preserve the published version of record.

**Figure:**