# Correction to: Synergistic Chemo-/Photothermal-therapy Based on Supercritical Technology-assisted Chitosan-Indocyanine Green/Luteolin Nanocomposites for Skin Wound Healing

**DOI:** 10.1093/rb/rbaf085

**Published:** 2025-09-04

This is a correction to: Pei-Yao Xu, Ranjith Kumar Kankala, Yue-Wei Li, Shi-Bin Wang, Ai-Zheng Chen, Synergistic chemo-/photothermal therapy based on supercritical technology-assisted chitosan–indocyanine green/luteolin nanocomposites for wound healing, *Regenerative Biomaterials*, Volume 9, 2022, rbac072, https://doi.org/10.1093/rb/rbac072

Figure 3 of this article contained an accidental typing error in FT-IR spectra, A corrected version of the figure and figure note appears below.


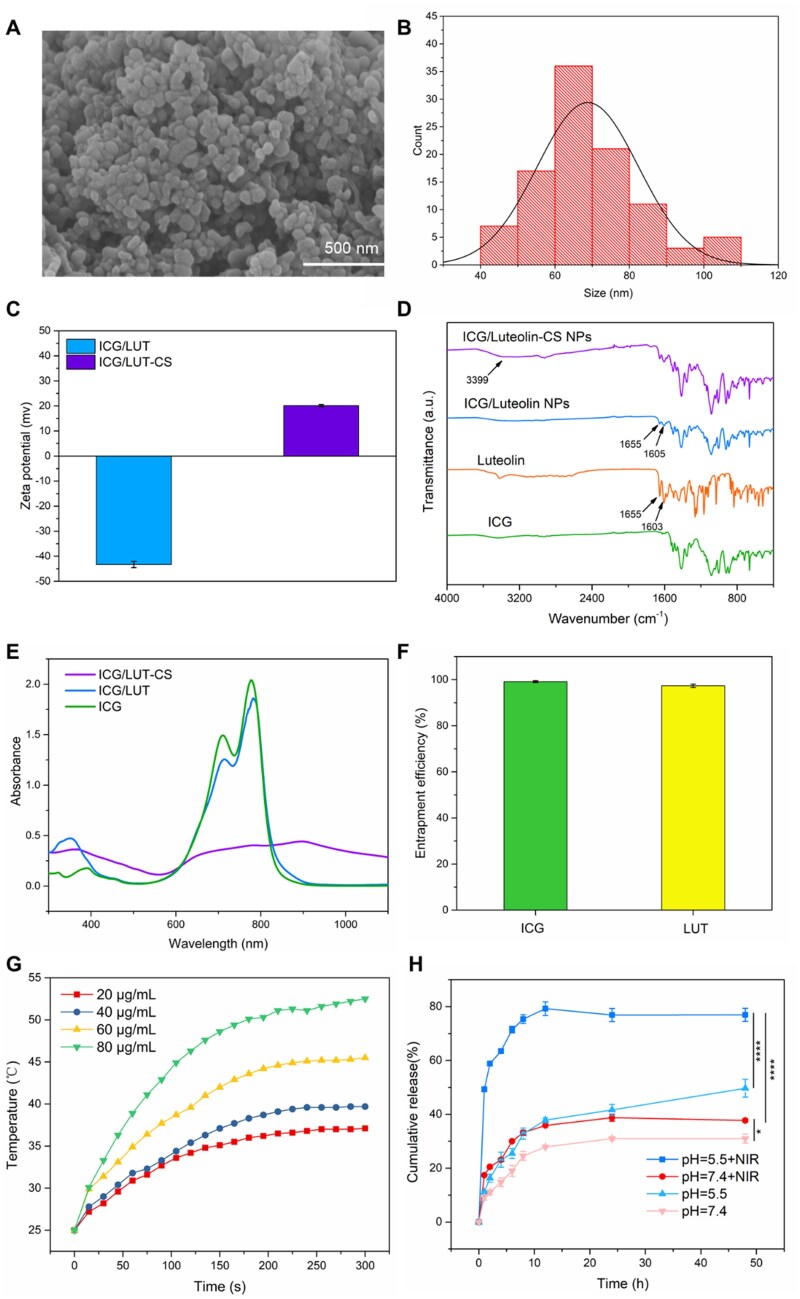


**Figure 3.** of this article contained an accidental merge error in the live/dead staining images of the remaining *S. aureus* biofilms with PBS treatment, as well as a typing error in the figure note. A corrected version of the figure and figure note appears below.


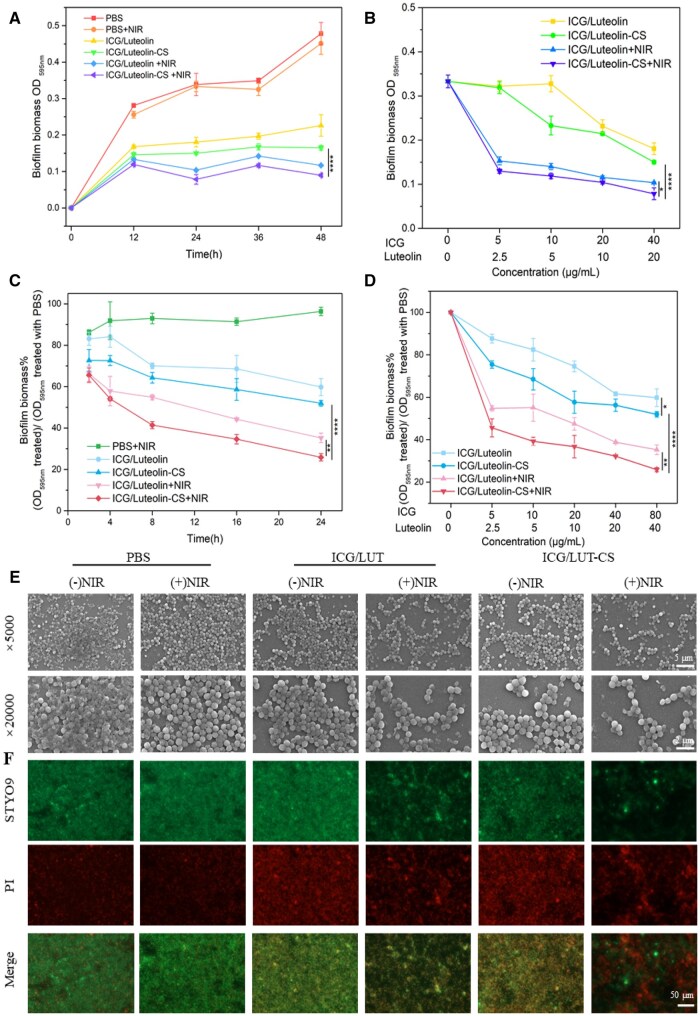


**Figure 5.**  *In vitro i*nhibition effect of *S.aureus* biofilm formation and *S.aureus* biofilm ablation performance of ICG/LUT-CS, (A) quantitative analysis of the CV-stained biofilms with different pretreatments for different times, (B) pretreatments with different concentrations of nanoparticles for 24 h, (C) relative the remaining *S.aureus* biofilm biomass with different treatment for different incubation times, (D) relative the remaining *S.aureus* biofilm biomass with different concentrations of nanoparticles for 24 h, (E) SEM images (scale bar=5 and 2 μm) and (F) live/dead staining images of remaining *S.aureus* biofilms with different treatments (scale bar=50 μm).


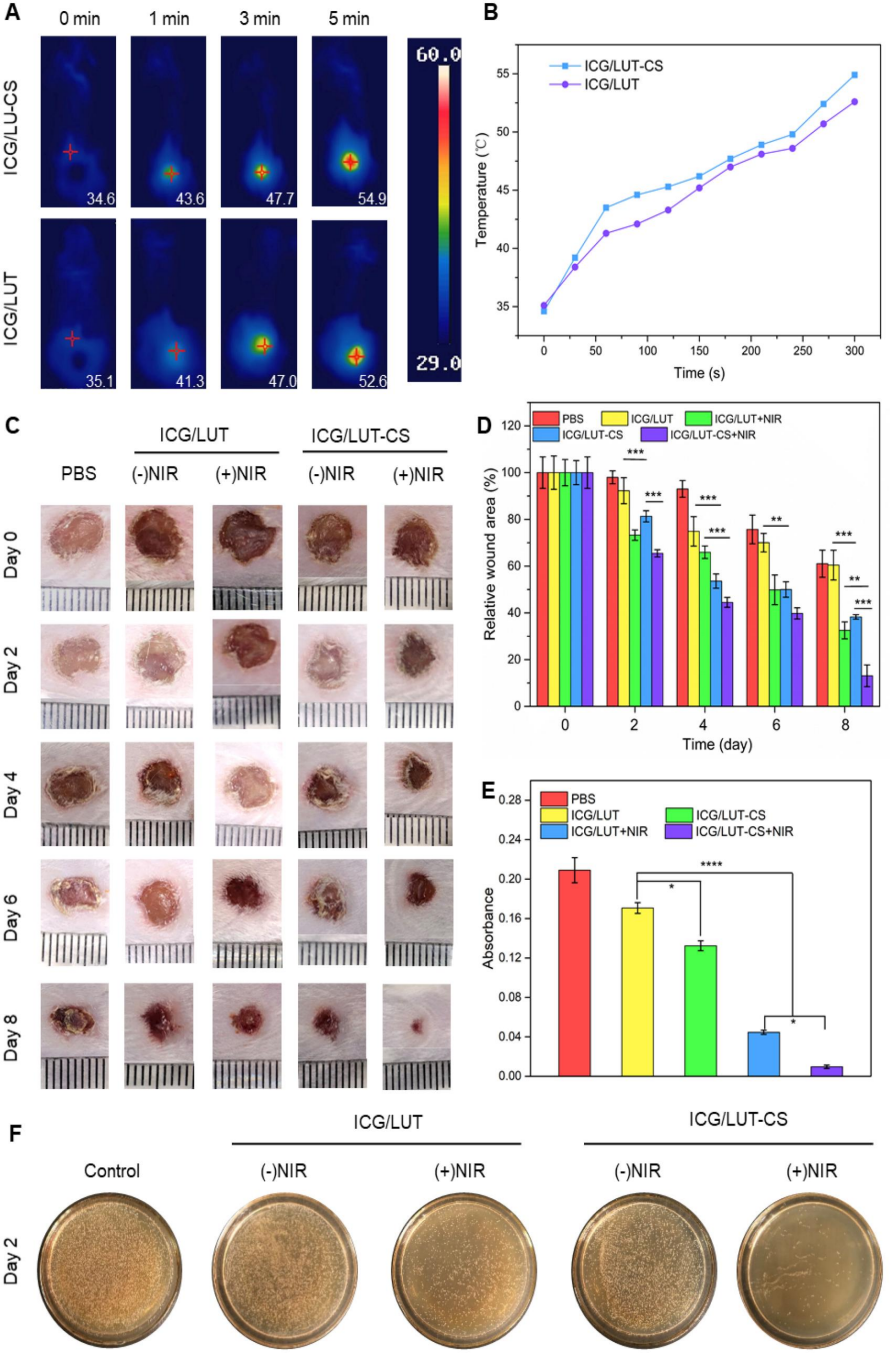
 **Figure 6.** of this article contained an accidental duplication in the representative images of wound images of (Day 2 ICG/Luteolin (+NIR)) and (Day 4 ICG/Luteolin (+NIR)). A corrected version of the figure appears below.

These details have been corrected only in this correction notice to preserve the published version of record.

