# Supplementary material for: Synergistic chemo-/photothermal therapy based on supercritical technology-assisted chitosan–indocyanine green/luteolin nanocomposites for wound healing
Source: Regen Biomater. 2022 Sep 26;9:rbac072. doi: 10.1093/rb/rbac072 (PMC9555995; doi:10.1093/rb/rbac072)
Supplement: rbac072_Supplementary_Data [file rbac072_supplementary_data.docx]

Supplementary data

**Synergistic Chemo-/Photothermal-therapy Based on Supercritical Technology-assisted Chitosan-Indocyanine Green/Luteolin Nanocomposites for Wound Healing**

**Pei-Yao Xu^1,2^, Ranjith Kumar Kankala^1,2^, Yue-Wei Li^1,2^, Shi-Bin Wang^1,2^, Ai-Zheng Chen^1,2,^***

^1^Institute of Biomaterials and Tissue Engineering, Huaqiao University, Xiamen, Fujian 361021, PR China.

^2^Fujian Provincial Key Laboratory of Biochemical Technology (Huaqiao University), Xiamen, Fujian 361021, PR China.

* Correspondence address. Institute of Biomaterials and Tissue Engineering, Huaqiao University, Xiamen 361021, PR. China. Emails: [azchen@hqu.edu.cn](mailto:azchen@hqu.edu.cn) (A. C.)

**Table S1.** Experimental results from the Minitab-based factorial design.

| **Run order** | **A**  **(Flow rate of CO_2_)** | **B**  **(Pressure)** | **C**  **(Flow rate of solution)** | **Average size of ICG/LUT (nm)** | **Span** |
| --- | --- | --- | --- | --- | --- |
| **1** | 40 | 12 | 1.00 | 68.4 | 0.84 |
| **2** | 30 | 10 | 1.00 | 72.7 | 0.72 |
| **3** | 30 | 12 | 0.50 | 82.5 | 0.63 |
| **4** | 40 | 12 | 0.50 | 49.5 | 0.65 |
| **5** | 35 | 11 | 0.75 | 76.9 | 0.68 |
| **6** | 40 | 10 | 0.50 | 105.4 | 0.76 |
| **7** | 30 | 12 | 1.00 | 52.7 | 0.79 |
| **8** | 35 | 11 | 0.75 | 80.1 | 0.66 |
| **9** | 40 | 10 | 1.00 | 75.7 | 0.55 |
| **10** | 30 | 10 | 0.50 | 123.1 | 0.70 |
| **11** | 35 | 11 | 0.75 | 77.3 | 0.72 |


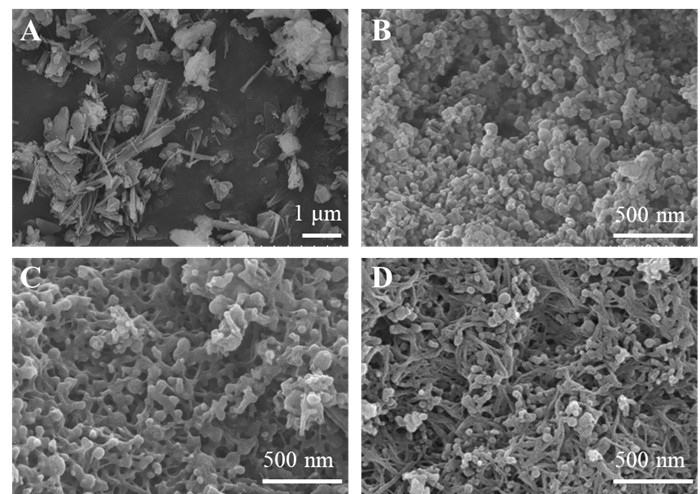


**Fig. S1** (**A**) SEM images of unprocessed LUT, (**B**) SEM images of ICG/LUT obtained by the SAS process at ICG/ LUT = 2:1, (**C**) 1:1, and (D) 1:2.


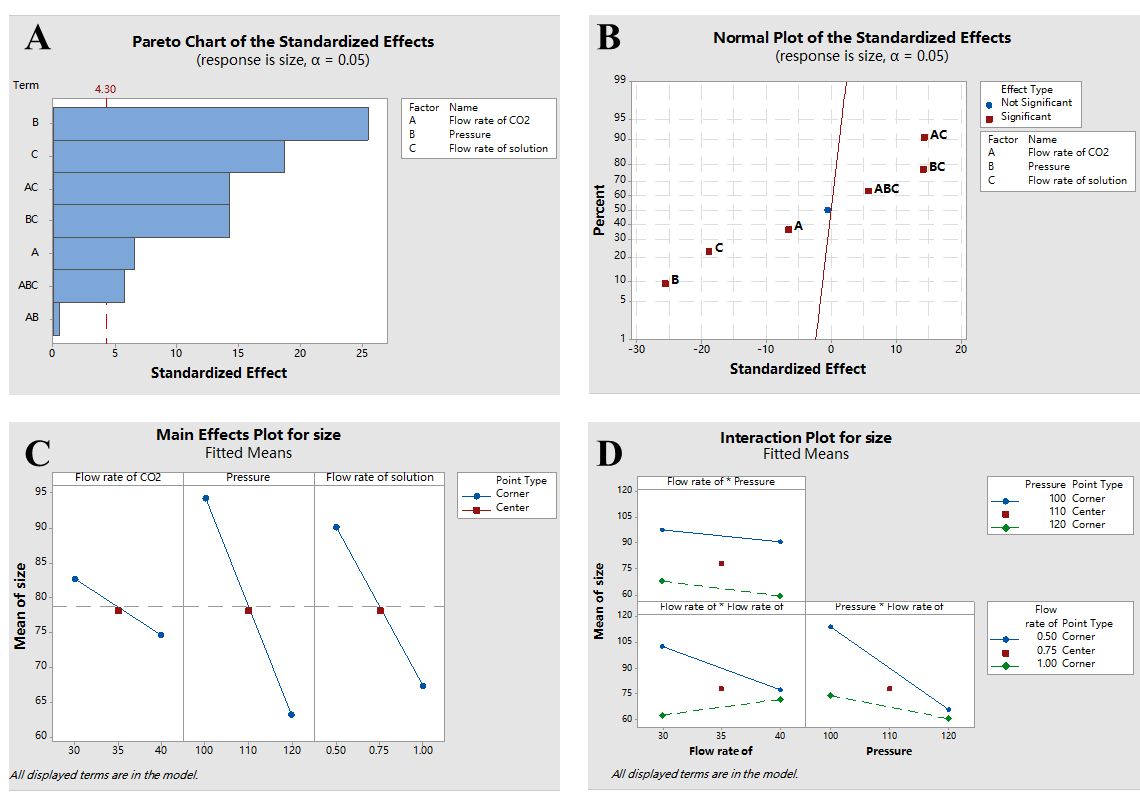


**Fig. S2** Theoretical assessment for the particle size of ICG/LUT. (**A**) Pareto chart of the standardized effects, (**B**) Normal probability plots, (**C**) Main effects plots, and (**D**) Interaction plot.


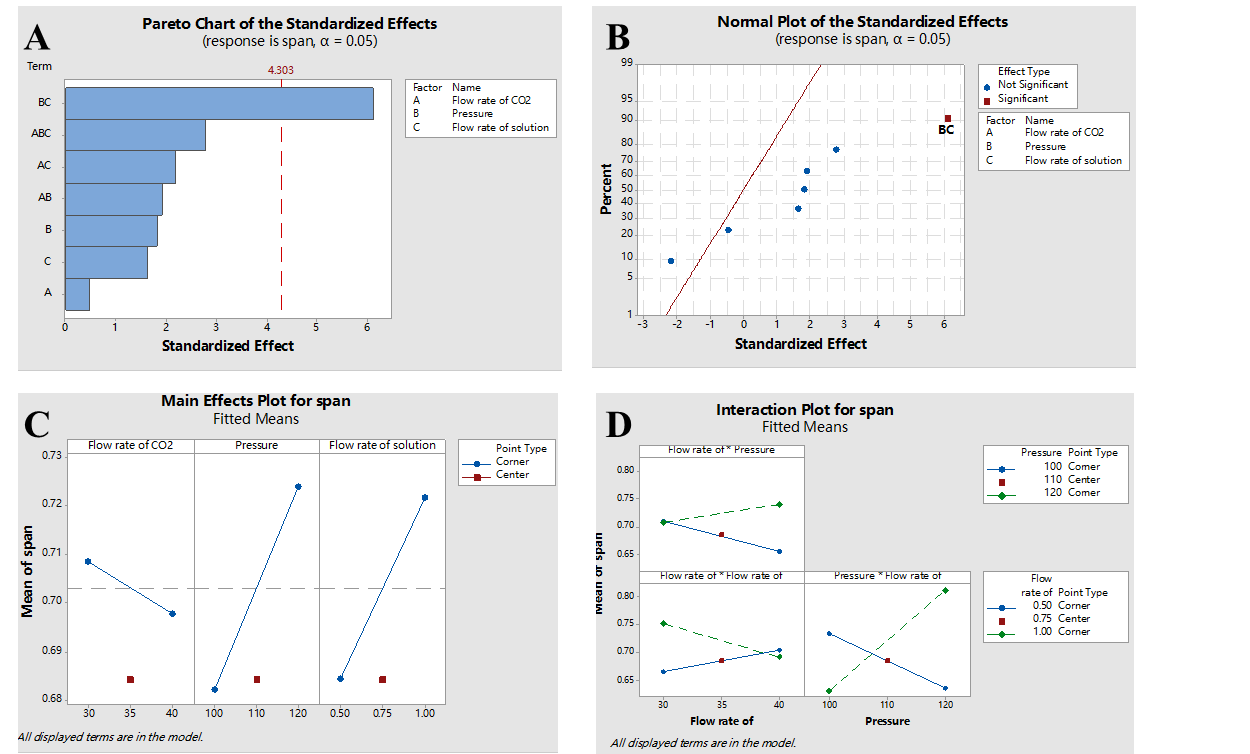


**Fig. S3** Theoretical assessment for the span of ICG/LUT. (**A**) Pareto chart of the standardized effects, (**B**) Normal probability plots, (**C**) Main effects plots, and (**D**) Interaction plot.

*
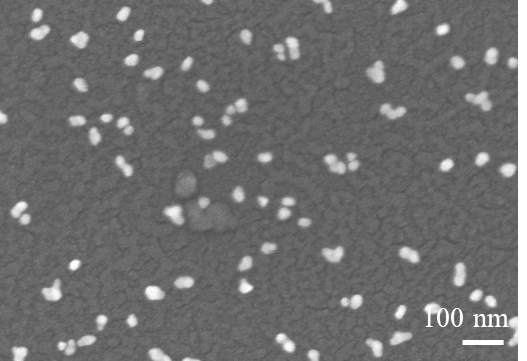
*

**Fig. S4** SEM image of ICG/LUT, scale bar = 100 nm.





**Fig. S5** XRD spectra of different samples.

*
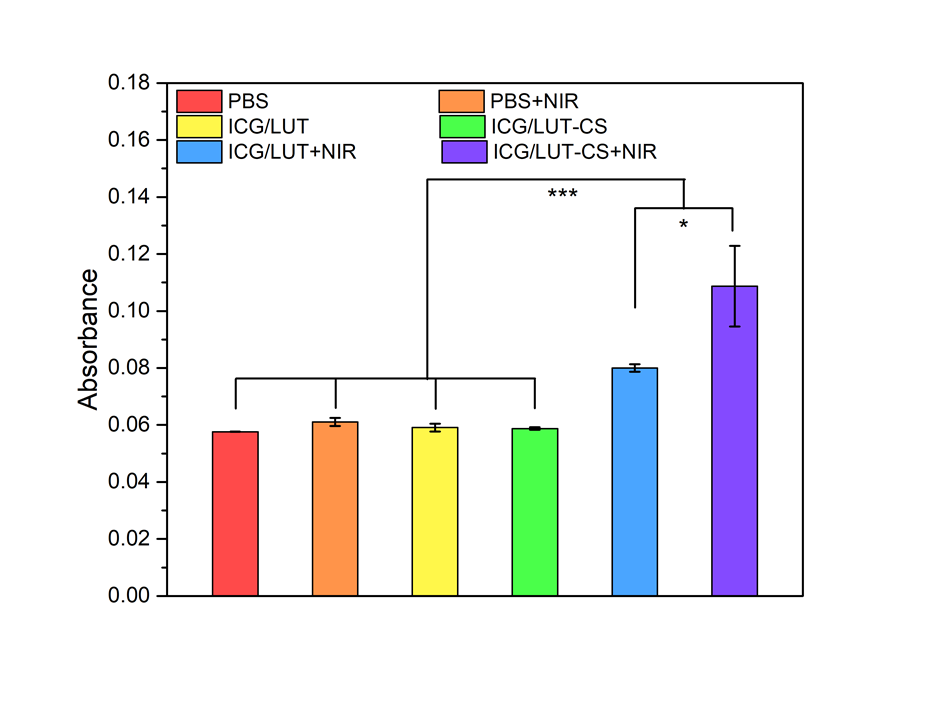
*

**Fig. S6** Membrane permeability of *S. aureus* detached from biofilm with different treatments by using ONPG hydrolysis assay.

*
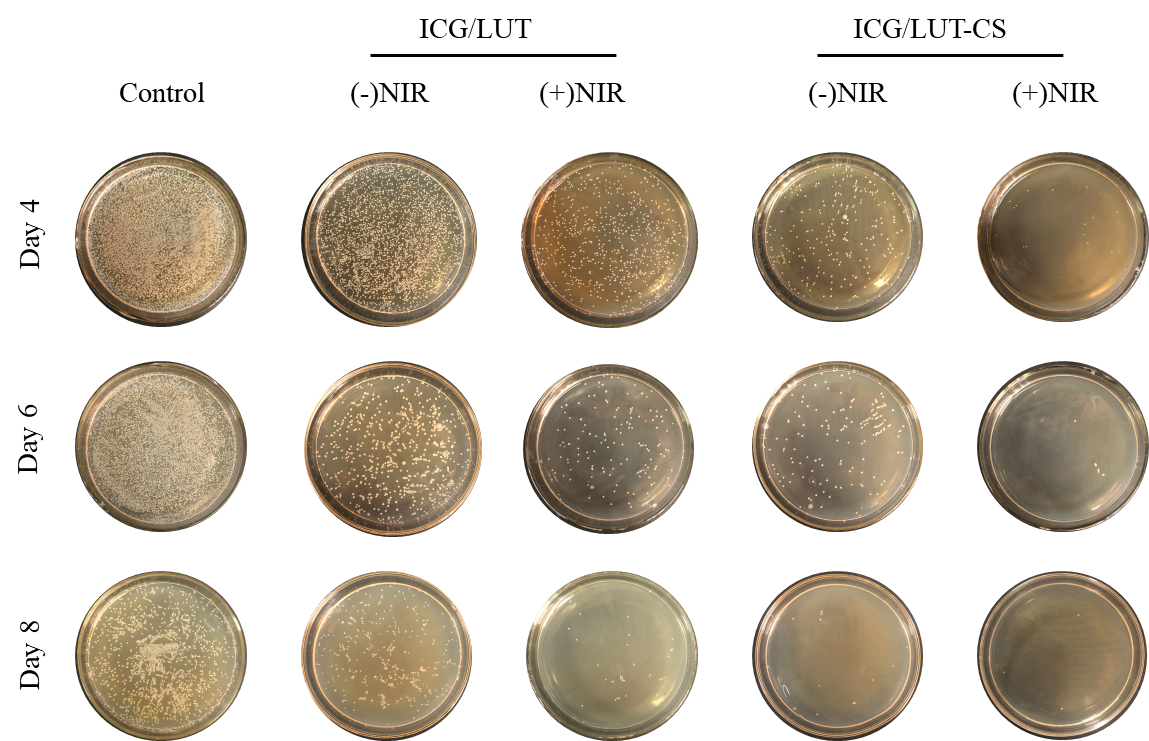
*

**Fig. S7** Photographs of *S. aureus* colonies isolated from wound site grown on broth agar plates after receiving various treatments for 4, 6, and 8 days.





**Fig. S8** Relative viabilities of NIH-3T3 cells after incubation with different samples at varying concentrations for 24 h after NIR treatment.

*
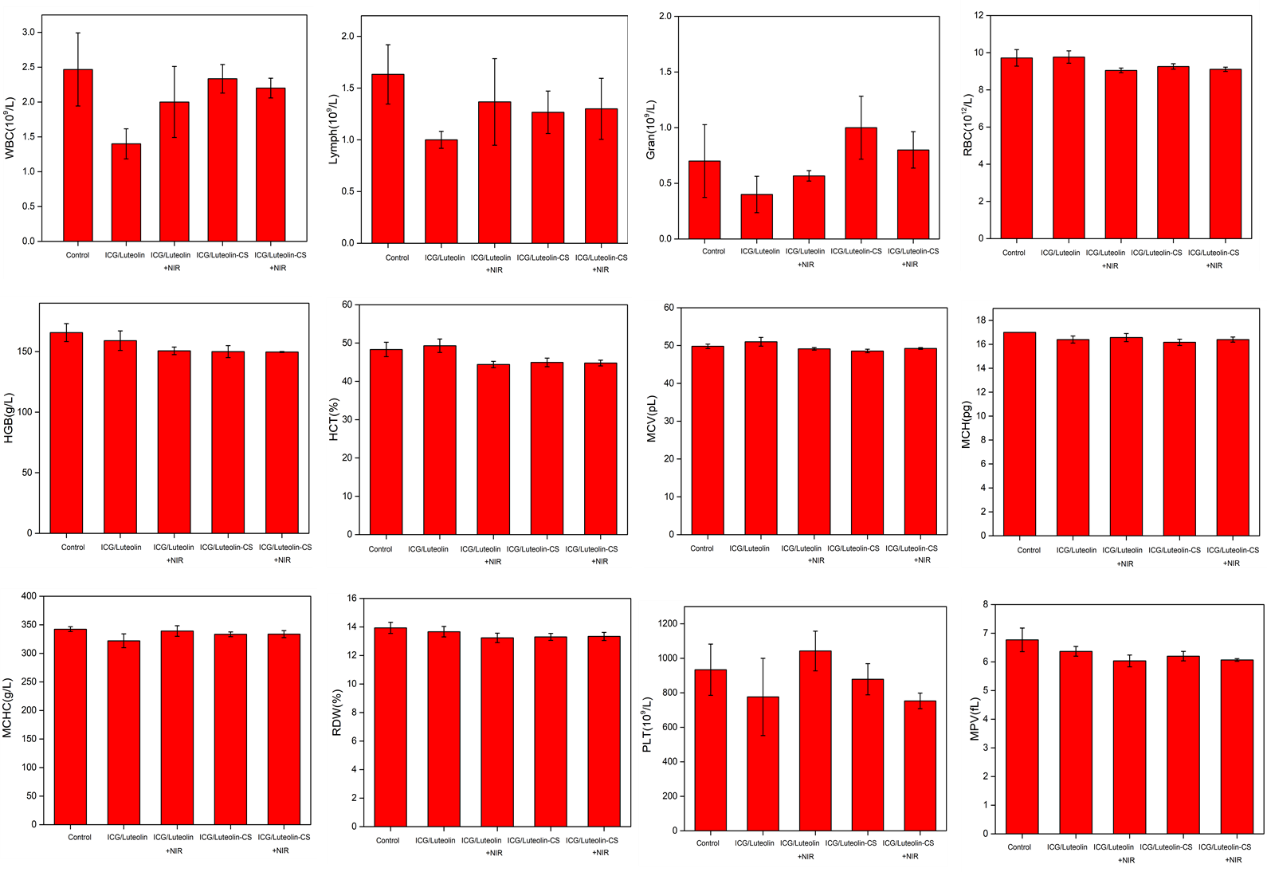
*

**Fig. S9** Levels of biochemical blood indexes after different treatments.
